# *Ligusticum sinense* Nanoemulsion Gel as Potential Repellent against *Aedes aegypti*, *Anopheles minimus*, and *Culex quinquefasciatus* (Diptera: Culicidae)

**DOI:** 10.3390/insects12070596

**Published:** 2021-06-30

**Authors:** Anuluck Junkum, Wanchai Maleewong, Atiporn Saeung, Danita Champakaew, Arpaporn Chansang, Doungporn Amornlerdpison, Arunee Kongdee Aldred, Udom Chaithong, Atchariya Jitpakdi, Doungrat Riyong, Benjawan Pitasawat

**Affiliations:** 1 Center of Insect Vector Study, Department of Parasitology, Faculty of Medicine, Chiang Mai University, Chiang Mai 50200, Thailand; atiporn.s@cmu.ac.th (A.S.); ar.chansang@gmail.com (A.C.); udom.c@cmu.ac.th (U.C.); ajitpakdi2@yahoo.com (A.J.); doungrat.riyong@cmu.ac.th (D.R.); benjawan.p@cmu.ac.th (B.P.); 2 Department of Parasitology and Excellence in Medical Innovation, and Technology Research Group, Faculty of Medicine and Mekong Health Science Research Institute, Khon Kaen University, Khon Kaen 40002, Thailand; wanch_ma@kku.ac.th; 3 School of Public Health, Walailak University, Nakhon Si Thammarat 80160, Thailand; danita.ch@wu.ac.th; 4 Center of Excellence in Agricultural Innovation for Graduate Entrepreneur, Maejo University, Chiang Mai 50290, Thailand; doungpornfishtech@gmail.com; 5 Program in Industrial Chemistry and Textile Technology, Faculty of Science, Maejo University, Chiang Mai 50290, Thailand; arunee.k@mju.ac.th

**Keywords:** DEET, *Ligusticum sinense*, mosquito, nanoformulation, repellent

## Abstract

**Simple Summary:**

Mosquito vectors can transmit diverse infectious pathogens and parasites that cause diseases such as dengue, Zika, Chikungunya, West Nile fever, and malaria. Botanical-derived repellents have been applied for personal protection against various species of mosquito vectors. In Thailand, numerous plant extracts have so far been investigated for mosquito repellent activity. Recently, *Ligusticum sinense* hexane extract (LHE) was reported as a potential candidate for the development of a new natural alternative to standard synthetic repellent. This study formulated LHE into nanoemulsion gel (LHE-NEG) and investigated its repellent activity against three mosquito vectors, including *Aedes aegypti*, *Anopheles minimus*, and *Culex quinquefasciatus*. The results revealed that LHE-NEG provided comparable protection times to N,N-diethyl-3-methylbenzamide nanoemulsion gel (DEET-NEG) against all mosquito species. Furthermore, LHE-NEG is safe for human health, based on the results of the skin irritation test. The repellent activity obtained from stored samples of LHE-NEG yielded satisfactory protection times of more than 2 h. Therefore, this gel formulation could be developed commercially, as an effective personal protection product against mosquito bites.

**Abstract:**

*Ligusticum sinense* Oliv. cv. is a species of Umbelliferae (Apiaceae), a large plant family in the order Apiales. In this study, *L. sinense* hexane extract nanoemulsion gel (LHE-NEG) was investigated for mosquito repellency and compared to the standard chemical, N,N-diethyl-3-methylbenzamide (DEET), with the goal of developing a natural alternative to synthetic repellents in protecting against mosquito vectors. The results demonstrated that LHE-NEG afforded remarkable repellency against *Aedes aegypti*, *Anopheles minimus*, and *Culex quinquefasciatus*, with median protection times (MPTs) of 5.5 (4.5–6.0), 11.5 (8.5–12.5), and 11.25 (8.5–12.5) h, respectively, which was comparable to those of DEET-nanoemulsion gel (DEET-NEG: 8.5 (7.0–9.0), 12.0 (10.0–12.5), and 12.5 (10.0–13.5) h, respectively). Evaluation of skin irritation in 30 human volunteers revealed no potential irritant from LHE-NEG. The physical and biological stability of LHE-NEG were determined after being kept under heating/cooling cycle conditions. The stored samples of LHE-NEG exhibited some changes in appearance and differing degrees of repellency between those kept for 3 and 6 heating/cooling cycles, thus providing slightly shorter MPTs of 4.25 (4.0–4.5) and 3.25 (2.5–3.5) h, respectively, when compared to those of 5.0 (4.5–6.0) h in fresh preparation. These findings encourage commercially developed LHE-based products as an alternative to conventional synthetic repellents in preventing mosquito bites and helping to interrupt mosquito-borne disease transmission.

## 1. Introduction

Mosquitoes are vectors of several communicable diseases such as dengue, malaria, filariasis, Japanese encephalitis, Zika, and others that are major health problems in tropical and subtropical countries. Over one million people worldwide die from mosquito-borne diseases each year [1]. Dengue and dengue hemorrhagic fever are transmitted by the bite of an infected *Aedes* mosquito, particularly *Aedes aegypti*. In 2019, the Department of Disease Control (DDC) from the Ministry of Public Health reported 128,401 dengue cases and 133 dengue deaths throughout Thailand [2]. No specific anti-viral drugs for treating this disease are available currently, and in this country a dengue vaccine has been licensed and approved only for use in people aged 9–45 years [3,4].

*Anopheles* mosquitoes are important vectors of malaria, which is a serious issue for society, causing mortality and morbidity as well as great financial loss. For the last several decades, malaria rates in Thailand have been decreasing dramatically, due to intensive vector control measures such as chemical insecticides and improved access to personal protection measures [5]. However, malaria cases are continually reported along border areas, especially between Thailand and Myanmar and Thailand and Cambodia. The problems in these areas are socioeconomic, healthcare infrastructure, and political situations associated with cross-border migration, which is difficult to manage appropriately [6].

*Culex quinquefasciatus* acts as a potential vector of Japanese encephalitis virus (JEV) and the filarial worm *Wuchereria bancrofti*, which is the agent of bancroftian filariasis in urban areas of Thailand [7,8]. Filariasis also causes long-term suffering and morbidity as well as high social and economic burden on individuals and communities. In Thailand, the number of migrant workers from endemic countries and hill tribes on the Thai–Myanmar border has been increasing over recent years [9,10]. This has prompted public health concerns about the possibility of increased transmission of bancroftian filariasis in Thailand.

The procedure for controlling or preventing the transmission of mosquito-borne diseases is to combat mosquito vector transmission by environmental management, chemical application (larvicide, adulticide), and personal protection from mosquito bites [11]. Regarding personal protection, N,N-diethyl-3-methylbenzamide (DEET) is the most common chemical compound used as an insect repellent by providing long-lasting protection against many mosquito species, ticks, chiggers, and other insects that transmit disease [12,13,14,15]. In addition, other chemical repellents such as dimethyl phthalate (DMP), para-methane 3-8, diol (PMD), ethyl butylacetylaminopropionate (IR3535), and Picaridin (KBR3023) have been reported as effective substances against mosquitoes and other biting insects [16,17]. DEET-based repellents are highly effective, but adverse reactions are associated with their application such as hives, redness, and irritation. Toxic reactions from DEET, which occur after misuse or prolonged exposure to high concentrations, also have been documented [12,14]. Furthermore, this compound has an unpleasant odor, oily feel, and high skin penetration, and it can damage plastic and synthetic fabric [18].

Most botanical-based repellents currently on the market contain extracts or essential oils from one or more of the following plants: Citronella (*Cymbopogon nardus*), cedar (*Juniper virginiana*), eucalyptus (*Eucalyptus maculate*), geranium (*Pelargonium reniforme*), lemon-grass (*Cymbopogon excavates*), peppermint (*Mentha piperita*), neem (*Azadirachta indica*), and soybean (*Neonotonia wightii*) [12,19]. However, these repellents may need frequently repeated application [20], as they provide short-lived efficacy when compared with synthetic chemicals. Therefore, the development of controlled release formulations such as cream, gel, lotion, polymer mixtures, or microcapsules are important in prolonging repellency of plant-derived products [21,22,23].

*Ligusticum sinense* Oliv. cv. is a species of Umbelliferae (Apiaceae), a large plant family in the order Apiales. Essential oils and phytochemicals isolated from several Umbelliferae plants have been reported as potential alternatives to conventional synthetic insecticides [22,24,25,26]. However, there have been few studies of *L. sinense* toxicity against pests of public health importance, particular mosquito vectors. Repellency screening of 15 medicinal plants demonstrated that *L. sinense* hexane extract (LHE) possesses the most promising efficacy against laboratory-reared *Ae. aegypti*, with a median complete-protection time of 6.5 (5.0–8.0) h [27]. Ethanol preparations of LHE also afforded remarkable repellency, with median complete-protection times of 11.5 (9.0–14.0) h and 6.5 (5.5–9.5) h against *Anopheles minimus* and *Ae. aegypti*, respectively [5]. Correspondingly, comparable repellency was achieved from DEET-ethanol formulations with median complete-protection times of 11.5 (10.5–15.0) h and 8.0 (5.0–9.5) h against *An. minimus* and *Ae. aegypti*, respectively. Gas chromatography–mass spectrometry (GC-MS) analysis of LHE revealed that the presence of 18 bioactive compounds accounted for 69.81% of all extract compositions, with the major components being 3-N-butylphthalide (31.46%), 2,5-dimethylpyridine (21.94%), and linoleic acid (16.41%) [5]. LHE, with its proven efficacy in repelling mosquitoes, is considered a potential candidate for developing new natural commercial products against mosquitoes. Herein, LHE gel nanoformulation was prepared and then evaluated for repellent efficacy in comparison to the gold standard (DEET). In addition, skin irritation and physical and biological stability were investigated.

## 2. Materials and Methods

### 2.1. Plant Materials and Extractions

Rhizomes of L. sinense were purchased from a supplier of medicinal herbs in Chiang Mai province, northern Thailand. The plant material was identified taxonomically by a scientist named Miss Wannaree Charoensup, from the Department of Pharmaceutical Science, Faculty of Pharmacy, Chiang Mai University (CMU), Chiang Mai, Thailand. The voucher specimen (PARA-LI-001-Rh/7) was placed at the Department of Parasitology, Faculty of Medicine, CMU. The L. sinense rhizomes were shade-dried at ambient temperature (30 ± 5 °C) in indoor conditions before being powdered by an electric grinder. Extraction was carried out on half a kilogram of dried powdered L. sinense rhizomes by macerating in 5 liters of hexane (VWR International Ltd, Leicestershire, UK) at room temperature (27 ± 5 °C) for at least 5–7 days until exhaustion of all extractable constituents. A Bücher funnel and Whatman No. 1 filter paper were used to filter the extracted mixtures, which were subsequently concentrated under reduced pressure at 40 °C on a rotary evaporator (EYELA, Tokyo, Japan). The liquid extract, with a light-brown color and aromatic odor characteristic of L. sinense, was obtained from residue freeze-dried at −55 °C and stored at −20 °C in air-tight brown bottles until used for repellent evaluation. 

### 2.2. Mosquito Test Populations 

The free-mating laboratory *An. minimus*, *Ae. aegypti*, and *Cx. quinquefasciatus* were maintained continuously in standard conditions (27 ± 2 °C, 85% ± 5% relative humidity (RH) and 14:10 h light/dark photoperiod cycle) without exposure to any insecticides or pathogens at the Department of Parasitology, Faculty of Medicine, CMU. Adults were given 10% sucrose solution supplemented with 10% multivitamin syrup ad libitum. Artificial membrane feeders with minor modifications [28] were used periodically to provide blood to females for egg maturation. Five to seven-day-old unfed females were used to evaluate repellent activity, and sugar-starved for 12 h before testing, to induce blood feeding during repellent investigations.

### 2.3. Human Volunteers

The participants comprised of six adult human volunteers (3 females and 3 males), who had no records of dermatological disease or allergies from arthropod bites, stings, or repellents. They were selected for this study from graduate students at CMU. After the participants took an interview and were advised comprehensively on the test objectives and methodology, possible discomforts related to repellency testing, and remedial arrangements, they signed an informed consent form. These volunteers were also asked to abstain from alcohol and avoid using scented products such as perfumes, colognes, deodorants, and lotion throughout the experimental period.

### 2.4. Preparation of Nanoemulsion (NE)

The formulation processes in preparations of *L. sinense* hexane extract nanoemulsion (LHE-NE) and DEET-nanoemulsion (DEET-NE) were similar, except for the active ingredient (LHE/DEET). Nanoemulsion was produced by a high-energy preparation method [29] with slight modifications. The formulation comprised several ingredients, including LHE/DEET, fixative (vanillin), surfactant (Tween^®^ 20), co-surfactant (glycerol), fragrance, and double deionized water. Coarse emulsion was prepared initially by adding double distilled water to the organic phase containing 40% LHE or 40% DEET and Tween^®^ 20:glycerol at a ratio of 5:1, as well as 10% vanillin and fragrance using a vortex mixer for 10 min. Then, the coarse LHE/DEET emulsion was subjected to ultrasonic emulsification using a 40-kHz sonicator (Drawell, Shanghai, China), with a power output of 120 watts at 25 °C for 2 h. After that, the formulated nanoemulsion was stored at 4 °C for LHE/DEET- nanoemulsion gel preparation. 

### 2.5. Preparation of Nanoemulsion Gel (NEG)

The 5% gelling agent was prepared by dissolving Carbopol^®^ 940 in double deionized water, mixing well by using a mixer and was left overnight for gel setting. The LHE-NE or DEET-NE was incorporated into the gel, followed by the addition of double deionized water. Then, humectant (propylene glycol), preservative (methyl- and propylparaben), and chelating agent (ethylenediaminetetraacetic acid: 1% EDTA) were added sequentially into the incorporated gel. Finally, triethanolamine (TEA) was added for pH adjustment. The formulated nanoemulsion gel was stirred by a mixer after each addition until well blended. The final concentration of LHE-NE/DEET-NE was 16% in the formulation. The compositions (all ingredients and their content) of nanoemulsion gel formulations of LHE (LHE-NEG) and DEET (DEET-NEG) are demonstrated in Table 1. Either LHE-NEG or DEET-NEG was packed in a suitable container, and kept at 4 °C as a further laboratory repellent bioassay against the three mosquito species (*Ae. aegypti*, *An. minimus*, and *Cx. quinquefasciatus*).

### 2.6. Evaluation on Repellent Activity of LHE-NEG and DEET-NEG Formulations

The LHE-NEG and DEET-NEG formulations were evaluated for repellency against various mosquito vectors, including *Ae. aegypti*, *An. minimus*, and *Cx. quinquefasciatus*, with the protocol modified from the World Health Organization (WHO) standard method [30], with volunteers of both sexes. As each mosquito species has a preference in biting, night-biting in *An. minimus* and *Cx. quinquefasciatus* were tested from 18.00 to 08.00 h, while *Ae. aegypti*, a day biter was tested between 06.00 to 18.00 h.

A total of 250 blood-starved female mosquitoes were selected randomly, transferred to each mosquito cage, and left to acclimatize for 1 h. Both arms of the volunteers were washed with distilled water, with rubber gloves covering the hands, and allowed to air dry. Each forearm of the volunteers was wrapped in a plastic sleeve, with a 30-cm^2^ (3 × 10 cm) rectangular portion cut out on the ventral part as a test or control area, thus exposing only the treated area to mosquitoes. With the help of a spatula, approximately 0.1 mg of each NEG formulation was applied to the exposed skin on one forearm. Absolute ethanol was applied on the other forearm to act as a control by using the same protocol as that performed on the NEG formulation-treated arm. Each trial started with the control arm being introduced into a mosquito cage and left there for 3 min. If 10 mosquitoes or more landed on or bit the control arm, the repellent testing continued, with the NEG formulation-treated forearm being exposed in a similar way to that of the control. Both the control and treated arms replaced each other regularly in order to verify and standardize the bite readiness of the mosquitoes and avoid bias. Complete protection time was determined once the treated forearm had been exposed for 3 min at 30-min intervals up until 2 bites occurring in a single period of exposure or 1 bite transpiring in each of two subsequent periods. Each test was replicated in all of the participants, with a new group of mosquitoes used on various days, and no volunteers were tested with more than one sample batch each day. Therefore, 12 replicates for each sample test were made with each volunteer being tested twice. The participants were blinded and assigned randomly to determine the sequence of their tests and treatments. Any undesirable effects such as skin irritation, burning sensation, unpleasant smell, etc. were recorded.

### 2.7. Evaluating Potential Skin Irritation from LHE-NEG and DEET-NEG 

The human 4-h patch test [31] was performed for evaluating skin irritant from LHE-NEG and DEET-NEG, using the commercially available patch; allergEAZE^®^ patch test chamber (SmartPractice^®^, Phoenix, AZ, USA). Thirty healthy male and female volunteers, aged 23–65 years and showing no signs of dermatological diseases were recruited as participants in this experiment. An allergEAZE^®^ patch test chamber was involved in the patch test procedure of applying 0.02 mg of NEG formulations onto the skin. The NEG-impregnated patch was put onto the skin of the inner upper arm of the participants for up to 4 h. The reference for negative and positive control groups was absolute ethanol and 20% aqueous sodium lauryl sulfate (20% SLS), respectively. The test patch was applied gradually from 15 to 30 min through 1, 2, 3, and 4 h, in order to avoid unacceptably high reactions. Shorter periods of exposure could be ignored if no overreaction occurring after longer exposures satisfied the study directors. In the 4-h period of exposure, the patch was taken off the skin, and the application site wiped softly with wet gauze and then rinsed with water. The applied sites were examined and scored at 24, 48, and 72 h after patch removal. Response to irritation was measured by using the four-point scale of increasing severity by Basketter et al. [31]: (0) No reaction; (+) weakly positive reaction, i.e., mild erythema or dryness across most of the treatment site; (++) moderately positive reaction, i.e., usually distinct erythema possibly spreading beyond the treatment site; and (+++) strongly positive reaction, i.e., strong, often spreading erythema with oedema. A volunteer with “+” or a greater reaction at any of the assessments was considered to have shown a positive skin irritant reaction.

### 2.8. Testing the Physical and Biological Stability of LHE-NEG

The samples of LHE-NEG were determined for physical characteristics and repellent activity against *Ae. aegypti* after they had been kept under a heating/cooling cycle for 3 and 6 cycles (1 cycle: Heated at 45 °C for 48 h and cooled at 4 °C for 48 h), and compared with fresh preparation. The test for repellent activity followed modification of the WHO standard method [30], as described previously. The repellent test was conducted twice on each of the six human volunteers.

### 2.9. Data Processing and Analysis

The median complete-protection time was used as a standard criterion for the repellency of the tested samples against *Ae. aegypti*, *An. minimus*, and *Cx. quinquefasciatus*. Repellent efficacy among the tested samples of each mosquito species was compared using the Mann–Whitney U test. The Kruskal–Wallis one-way ANOVA was used to compare the repellent activity between fresh and stored samples of LHE-NEG. Statistical analyses were conducted using IBM SPSS statistics, version 22 for Windows (Armonk, NY, USA). Statistical significance was set at *p* < 0.05.

## 3. Results

### 3.1. Repellent Activity of LHE-NEG

The average size of LHE-NE and DEET-NE was 169.2 (161.8–173.4) d.nm and 8.635 (8.516–8.794) d.nm, respectively, with a polydispersion index (PdI) of 0.25 and 0.19, respectively, as measured using a Zetasizer. Results in Table 2 demonstrate that LHE-NEG was effective prominently in repelling *Ae. aegypti*, *An. minimus*, and *Cx. quinquefasciatus*. The median complete-protection times provided by LHE-NEG and DEET-NEG against *Ae. aegypti*, *An. minimus*, and *Cx. quinquefasciatus* were 5.5 (4.5–6.0) h and 8.5 (7.0–9.0) h, 11.5 (8.5–12.5) h, and 12.0 (10.0–12.5) h, and 11.25 (8.5–12.5) h and 12.5 (10.0–13.5) h, respectively. There were significant differences between the repellency of LHE-NEG and DEET-NEG against *Ae. aegypti* (Z = −4.213, *p* < 0.0001) and *Cx. quinquefasciatus* (Z = –2.068, *p* = 0.039), but no significant differences against *An. minimus* (Z = –1.565, *p* = 0.118). No skin irritation, rashes, swelling, or other allergic responses were observed during the study period.

### 3.2. Skin Irritant Potential of LHE-NEG and DEET-NEG

Results from the skin irritation test stated that none of the 30 participants, who partook in the 4-h patch test, showed a positive skin irritant reaction to LHE-NEG or DEET-NEG in any of the evaluations (Table 3). Similar results were obtained from the application of absolute ethanol, which was a negative control reference. In contrast, six volunteers presented with a positive irritant reaction to a positive control reference of 20% sodium lauryl sulfate (20% SLS), which is an anionic surfactant used commonly in cosmetics as an emulsifying agent. Nine adult volunteers showed positive irritant with slight (+) reactions at 24 h after SLS-patch removal. In addition, slight (+) and moderate (++) irritant reactions were also observed in five and one of the volunteers, respectively, at 72 h after SLS-patch removal. 

### 3.3. Physical and Biological Stability

Appearance, physical properties, and repellency of the stored LHE-NEG against *Ae. aegypti* mosquitoes are exhibited in Table 4. After being kept under heating/cooling cycles for 3 and 6 cycles, all heated and cooled products of LHE-NEG were soft gel, with a pleasant aromatic odor, similar to that of the fresh preparation, whereas the color of the samples changed from whitish cream to pale yellow at six cycles. The LHE-NEG samples exhibited repellent activity against *Ae. aegypti* with the median complete protection time of 3.25 h (2.5–3.5 h) when kept under six heating/cooling cycles, which was significantly shorter than that of the fresh preparation (5.0 h, 4.5–6.0 h) (*p* < 0.0001) that kept under three heating/cooling cycles (4.25 h, 4.0–4.5 h) (*p* = 0.004). There were no significant differences regarding repellency between the fresh and three heated/cooled products of LHE-NEG (*p* = 0.101). 

## 4. Discussion

In general, a key feature of commercially-registered alternative insect repellents is the superiority or equivalent repellency to conventional products. Formulation and preparation by enhancing efficiency, safety, and stability is a significant step in obtaining proper and practical repellents. Therefore, formulated preparation is required to fix and control the release of plant compounds, especially active ingredients, resulting in improved protection against mosquitoes and other insects. Up until now, several researchers have reported the use of the sustained-release technology, nanoemulsions, as a suitable carrier of active essential oils against mosquito vectors [22,23,32,33].

Due to the impressive repellency of LHE against both *Ae*. *aegypti* and *An*. *minimus* [5,27], this product was subjected to further preparation in this study as the nanoemulsion gel, LHE-NEG. The results obtained from repellent activity demonstrated pronounced efficacy of LHE-NEG against laboratory-reared *Ae*. *aegypti*, *An*. *minimus*, and *Cx*. *quinquefasciatus*. The protection of formulated nanoemulsion was due presumably to the small droplet sizes of LHE-loaded nanoemulsion, which increased the surface area of droplets, thereby, increasing repellent activity. The findings in this study were in accordance with those of Nuchuchua et al. [32], who reported that a small nanoscale of essential oil-loaded nanoemulsion, prepared by high-pressure homogenization, would play an important role in repellent efficacy. The nanodroplets could be well formed and spread on the skin’s surface as a thin film, resulting in the increased vaporization of essential oils and subsequently, prolonged mosquito repellent activity. The improved repellency obtained from LHE-NEG, which was prepared in gel formulation, may be attributed to the effect of the gelling agent, Carbopol^®^ 940, which may conserve persistence of the active ingredient by making the gel-film a tightly coated skin surface. Similar to the study of Tuetun et al. [34], which revealed that repellency against *Ae*. *aegypti*, afforded by formulated gels (2–4.5 h) of *Apium graveolens*, mostly appeared to have higher repellency than formulated solutions (0–3.5 h). The G10 gel-formulation, which comprises *A*. *graveolens* hexane extract, orange oil, eucalyptus oil, vanillin, Carbopol^®^ Ultrez 21, propylene glycol, preservative solution, deionized water, D-ethanol, 50% neutral TE, and PER-RH 40 was the best formula, with the longest-lasting protection time of 4.5 h (4.5–5.0 h). In this study, other substances that were formulated in LHE-NEG such as TEA, propylene glycol, methyl- and propylparaben, and EDTA, which are used commonly as a neutralizer, humectant, preservative, and chelating agents, respectively, might be influential in enhancing effectiveness in repelling mosquitoes, presumably by synergistic and/or other properties. However, the slightly lower repellency of LHE-NEG (LHE = 16%), when compared to its ethanolic preparations (LHE = 25%) [5], is due possibly to the reduced content of active ingredients. In addition, unstable efficacy that is found commonly in botanical active ingredients [5], might be responsible for repellent efficacy. This aspect needs further investigation for clarification. 

The repellency of LHE-NEG against *Ae. aegypti* and *Cx. quinquefasciatus*, observed herein, was lower than that against *An. minimus*. These findings are supported by the study of Cilex et al. [35], which indicated that *Ae. aegypti* was the most aggressive host-seeking mosquito species because it has primarily an anthropophilic preference, whereas *An. minimus* s.l. and *Cx. quinquefasciatus* show ornithrophilic and zoophilic preference by feeding on humans only occasionally [36,37,38]. Afify and Potter [39] stated correspondingly that different species of mosquitoes have different behavioral responses to repellents. They suggested further that the olfactory systems of anophelinae and culicinae evolved independently and responded differently to odorants, including repellents. Amer and Mehlhorn [40] revealed that the sensillum numbers and distribution patterns between culicine and anopheline mosquitoes on maxillary bulbs play a role in the perception of repellent. Stanczyk et al. [41] also reported that *Ae. aegypti* decreased in behavioral repellency after 3 h of exposure to DEET, due to change in the olfactory system and also adaptive behavior.

The skin irritant test showed that topical application of LHE-NEG did not irritate human skin. The result obtained showed strong supportive evidence that established the safety of LHE for its proposed applications to human volunteers in the mosquito repellent study. Furthermore, no local skin reactions such as rash, swelling, irritation, burning sensation, or other allergic responses were observed in the volunteers during laboratory study periods. In addition, *L*. *sinense* (or *Ligusticum chuanxiong*) was proven to have an inhibited cell viability that induced cell apoptosis, particularly hypertrophic scar fibroblasts on human skin [42], which supported the relatively safe application of this plant product on the skin.

Prior to the introduction of any newly developed product as a potential repellent, it is necessary to determine their physical degradation property and biological stability, which can be affected by environmental factors such as temperature, pH, light, and humidity [43]. In this study, testing the heating/cooling cycle stability of gel nanoformulations was, therefore, conducted in order to assess their short-term physical and biological stability. After storage under three and six cycles of heating and cooling, it was found that the stored samples of this product exhibited some changes in color and varying degrees of repellency when compared to those of the fresh preparation. Turek and Stintzing [44] reported that temperature crucially affected stability of essential oil such as oxidative and polymerization processes, which lead to loss of quality and pharmacological properties. The effect of temperature on the duration of repellent efficacy of plant-based products, essential oils, and solvent extracts against mosquitoes has been reported by many researchers [5,45,46,47]. However, repellent activities offered from these LHE-NEG stored samples still produced satisfactory protection times (3.25–4.25 h), which exceeded the minimum requirement (2 h) of the Food and Drug Administration (FDA) for sale in Thailand. Consequently, LHE-NEG with proven repellent efficacy, no side effects on the skin, and a relatively stable physical and biological performance could qualify for development and registration as a new natural alternative to DEET.

## 5. Conclusions

Incorporating LHE using high-pressure homogenization to prepare LHE-NEG, a nanoemulsion repellent product, yielded impressive repellent activity against *Ae*. *aegypti*, *An*. *minimus*, and *Cx*. *quinquefasciatus*. Field evaluation on the repellency of LHE-NEG against a wide range of mosquito species will be carried out in further study.

## Figures and Tables

**Table 1:** Composition (%) of nanoemulsion gel formulations, LHE-NEG, and DEET-NEG.

Ingredients (g)	Formulations	
	LHE-NEG	DEET-NEG
LHE-NE	40.0	-
DEET-NE	-	40.0
5% Carbopol^®^940	17.5	17.5
Double deionized water	33.0	33.0
Propylene glycol	5.0	5.0
1% EDTA	3.0	3.0
Methylparaben	0.2	0.2
Propylparaben	0.1	0.1
TEA	1.2	1.2

**Table 2:** Repellent activity of LHE-NEG and DEET-NEG against female *Aedes aegypti*, *Anopheles minimus*, and *Culex quinquefasciatus*.

Repellent Samples	Median Complete Protection Time (Range, h) ^1,^*		
	*Ae*. *aegypti*	*An*. *minimus*	*Cx*. *quinquefasciatus*
LHE-NEG	5.5 (4.5–6.0) ^a^	11.5 (8.5–12.5) ^c^	11.25 (8.5–12.5) ^d^
DEET-NEG	8.5 (7.0–9.0) ^b^	12.0 (10.0–12.5) ^c^	12.5 (10.0–13.5) ^e^

^1^ There were 12 replicates of each test. * Values followed by different letters in a column were significantly different (Mann–Whitney *U* test, *p* < 0.05).

**Table 3:** Skin irritant potential of LHE-NEG, DEET-NEG, 20% SLS, and absolute ethanol.

Treatment	Number of Volunteers	Score for Skin Irritation ^1^			
		0	+	++	+++
LHE-NEG	30	30	0	0	0
DEET-NEG	30	30	0	0	0
20% SLS	30	24	5	1	0
Absolute ethanol	30	30	0	0	0

Each application site on the skin was examined and scored at 72 h after patch removal: ^1^ Number of volunteers whose highest score in these three evaluations was (0) absence of irritation, (+) slight irritation, (++) moderate irritation, and (+++) severe irritation.

**Table 4:** Appearance, physical characteristics, and repellency of LHE-NEG samples against *Aedes aegypti* after being kept under 0, 3, and 6 heating/cooling cycles.

LHE-NEG: Heating/Cooling Cycles	Appearance & Physical Characteristics			Median Complete Protection Time (Range, h) ^1,^*
	Consistency	Color	Odor	
0	Soft	Whitish cream	Aromatic	5.0 (4.5–6.0) ^a^
3	Soft	Whitish cream	Aromatic	4.25 (4.0–4.5) ^a^
6	Soft	Pale yellow	Aromatic	3.25 (2.5–3.5) ^b^

^1^ There were 12 replicates of each test. * Values followed by different letters in a column were significantly different (Kruskal–Wallis one-way ANOVA, *p* < 0.05).
